# A Novel Simulated Moving Plug Flow Crystallizer (SM-PFC) for Addressing the Encrustation Problem: Simulation-Based Studies on Cooling Crystallization

**DOI:** 10.1021/acs.iecr.2c02862

**Published:** 2023-03-10

**Authors:** Aaron Bjarnason, Aniruddha Majumder

**Affiliations:** School of Engineering, University of Aberdeen, Aberdeen AB24 3UE, U.K.

## Abstract

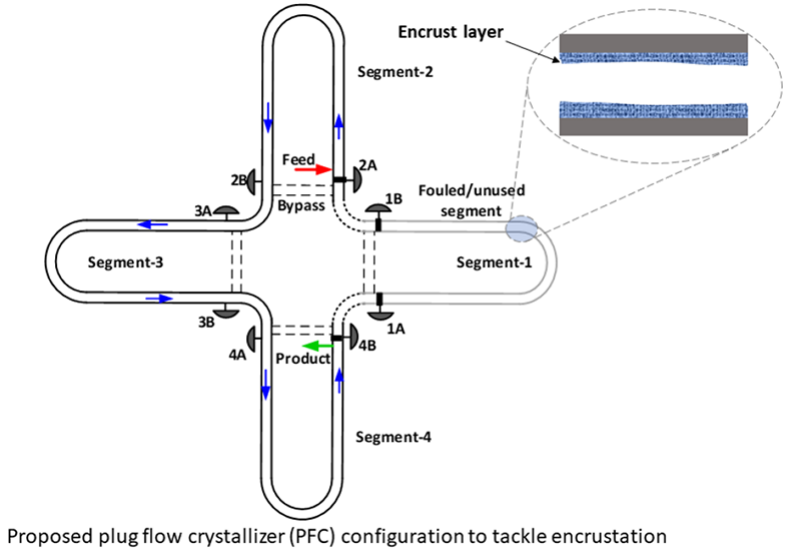

The plug flow crystallizer (PFC) is a promising candidate in the move toward adoption of continuous manufacturing in the pharmaceutical industry. However, a major concern for the smooth running of PFCs is the encrustation or fouling which can result in blockage of the crystallizer or unplanned shutdown of the process. In order to address this problem, simulation studies are carried out to explore the feasibility of a novel simulated-moving PFC (SM-PFC) configuration that can run uninterrupted in the presence of heavy fouling without compromising the desired critical quality attributes of the product crystals. The key concept of the SM-PFC lies in the arrangement of the crystallizer segments where a fouled segment is isolated, while a clean segment is simultaneously brought online avoiding fouling-related issues and maintaining uninterrupted operation. The inlet and outlet ports are also changed appropriately so that the whole operation mimics the movement of the PFC. The simulation results suggest that the proposed PFC configuration could be a potential mitigating approach for the encrustation problem enabling continuous operation of the crystallizer in the presence of heavy fouling while maintaining the product specifications.

## Introduction

The need to ramp up pharmaceutical production in situations such as a pandemic means that a more efficient and streamlined approach in pharmaceutical manufacturing is required to mitigate the potential shortage of lifesaving drugs. In recent years, the continuous plug flow crystallizer (PFC) such as the continuous oscillatory baffled crystallizer (COBC) has been identified as a promising candidate in improving pharmaceutical production processes due to several benefits such as improvement in processing time, product consistency, ease of scale up, and cost reduction. Despite the aforementioned benefits, there are challenges associated with the PFC, mainly centered around the issues of fouling or encrustation.^1^ The PFC is typically run in a steady-state condition where the liquid phase of the slurry is generally supersaturated. Such supersaturation can cause deposition of fouling on the interior surface of the PFC, in situ sensors and in the transfer lines leading to changes in the mean residence time, poor heat transfer, compromised product crystal quality, or even an abrupt shut down of the process.^2,3^ The lack of ability to run the PFC for extended periods without fouling can have a detrimental effect on some of the gains expected from continuous crystallization.^1^

Over the years, a good number of studies - both experimental and computational - on crystallizer fouling have been reported in the literature. Bohnet^4^ modeled the encrustation of CaSO_4_ in an aqueous solution by considering several stages, i.e., initiation, transport, attachment, removal, and aging, and showed a good agreement between the computational and experimental data. Brahim et al.^5^ combined the computational fluid dynamics (CFD) and encrustation model to study the fouling of aqueous CaSO_4_. However, due to the computational burden, only fictitious crystal growth was simulated. Zhang et al.^6^ later improved the model by combining the CFD with a pseudodynamic scheme, where the dynamic fouling process is approximated as a set of sequential steady-state processes taken place in a continuously varying geometric domain, to study the fouling of the CaSO_4_ solution. However, none of these studies consider the effect of encrustation on the crystal size distribution (CSD). Majumder and Nagy^7^ carried out a simulation study on how the encrustation affects the product CSD in a PFC by combining the encrust model, population balance model, mass, and energy balances.

The detection and monitoring of encrustation are critical as this can provide guidance on time when any mitigation strategies should be implemented. Various direct and indirect techniques are available for this purpose. The direct methods involve disassembling the fouled unit and taking measurements of the amount, thickness, and chemical composition of the fouling material.^8^ These methods can provide useful information to understand encrustation. However, they are not suitable for online monitoring. On the contrary, indirect methods are noninvasive and able to determine encrustation by studying its effect on process variables such as pressure drop,^9^ electrical resistance,^10^ thermal resistance,^11,12^ acoustic properties,^13^ and image processing.^8^ These noninvasive techniques will be useful for the online monitoring of the crystallization fouling in a PFC.

There are several encrustation mitigation strategies proposed over the years with their own advantages and disadvantages. One obvious strategy is to reduce the driving force for encrustation which is supersaturation. Lower supersaturation also affects the crystallization process adversely as the driving force for both the crystallization and encrustation is the same.^3^ Chemical additives have also been used to suppress the encrustation.^14^ The other usage of additives includes control of morphology or polymorphs during crystallization.^15,16^ However, in some studies, additives are found to suppress nucleation and crystal growth leading to a low yield of crystallization.^17^ Moreover, the use of additives increases the plant operating cost.^3^ Koswara and Nagy^18^ proposed encrust dissolution using temperature cycling along the PFC causing crystal growth and dissolution to occur in different segments. Such temperature cycling will also affect the mean crystal size of the product and can produce crystals smaller than the specified size range.^3^ Crystallizer surface modification is another approach to tackle encrustation.^19^ Wu et al.^20^ reported that encrustation on the crystallizer surface during the crystallization of indomethacin was significantly inhibited by nanocoating of gold (10 nm) and a polyelectrolyte. However, surface modifications can extend the induction period for the fouling (e.g., from 220%–453%), but it cannot get rid of the fouling process.^21,22^ Therefore, the crystallizer will still be subject to fouling after the induction period. Moreover, some coatings are prone to significant aging which leads to poor abrasion resistance.^23^

In this work, we propose intelligent equipment and process design strategies which do not require any additional additives and surface modification to be used and would not compromise the required critical quality attributes of the product crystals in the presence of heavy fouling. To the best of our knowledge, such a novel crystallizer platform has not been proposed before. The key idea here is to design the PFC platform such that the fouled segment of the PFC can be isolated and cleaned without interrupting the crystallization process. The details are discussed in the next section.

## Proposed Simulated Moving Plug Flow Crystallizer (SM-PFC)

**Figure 1:**
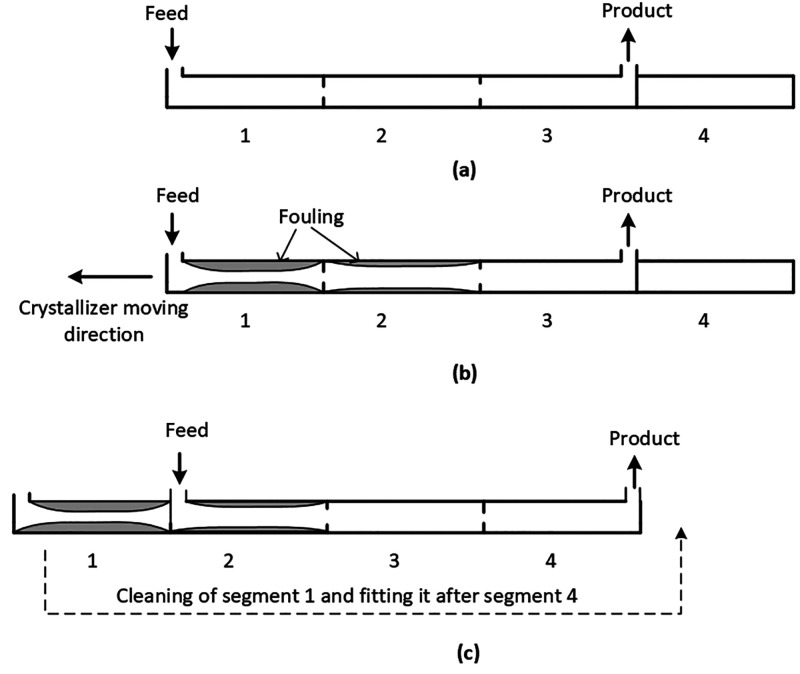
Schematic of a true moving plug flow crystallizer to mitigate the encrustation problem.

In order to understand the concept behind this simulated moving PFC (SM-PFC) platform, we will first consider a true moving plug flow crystallizer (TM-PFC). Let us assume that we have a PFC with four segments, of which three segments are used for crystallization at a given time. At the beginning, we use the first three segments in the crystallization process as shown in Figure 1, while the fourth segment is on standby for future use. After some time, the segments are subjected to fouling. Assuming a natural cooling profile in the PFC, the degree of fouling is higher in segment-1 as the driving force for fouling is larger in this segment where the feed first comes in contact with the cooling fluid. However, the concept explained here is applicable to other cooling profiles for which higher fouling can occur in other segments. If the first segment is isolated, the standby segment is appended at the end of the PFC, and the feed is introduced to the second segment by shifting the PFC to the left; then, we would be able to use the next three segments, i.e., 2–4, for crystallization. The fouled segment then can be cleaned e.g., by flowing hot solvent through it to dissolve the encrust layer and made ready for use by fitting it after segment-4 as shown in Figure 1(c). This can be called a true moving plug flow crystallizer (TM-PFC).

**Figure 2:**
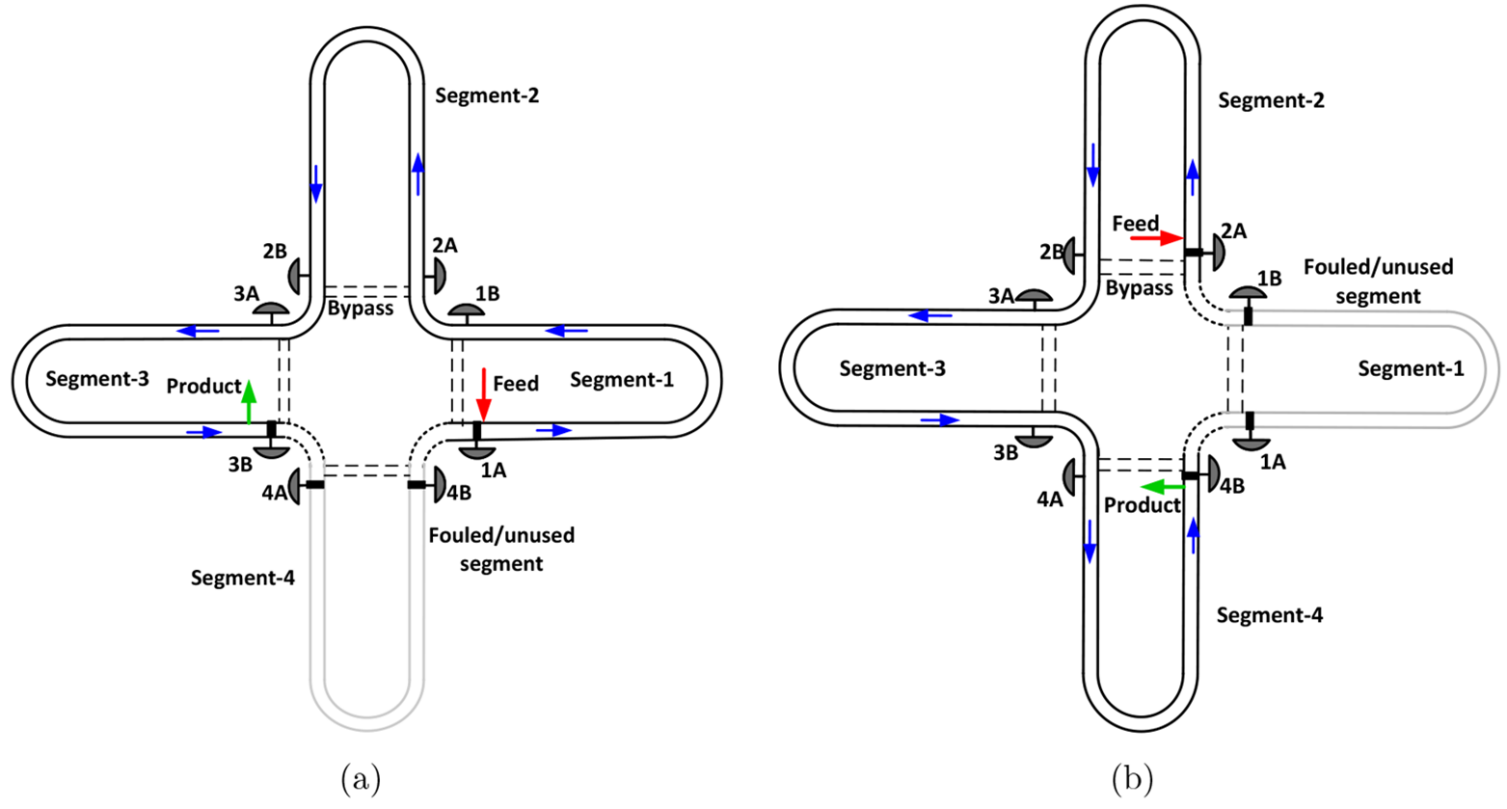
Schematic of the proposed SM-PFC configuration. (a) The feed port is at segment-1, and the outlet port is at segment-3. Segment-4 is unused. (b) When enough fouling is detected in segment-1, the feed is switched to segment-2, and the outlet is switched to segment-4, while segment-1 is isolated, cleaned, and prepared for next usage. This simulates movement of the crystallizer in a clockwise direction.

In practice, operating such a TM-PFC would be very inconvenient which requires manual disassembling and assembling of PFC segments at a regular time interval. However, we propose a hardware configuration which can simulate such movement in the crystallizer allowing enough time for the automated cleaning and reuse of the fouled segment without interrupting the crystallization process. The schematic of the proposed configuration is shown in Figure 2. In the proposed configuration, the four segments of a PFC are arranged in a star shaped structure. There are provisions for feed injection and product withdrawal in each segment. At a given time, only three segments are in use, while the fourth segment is at rest or being cleaned as shown in Figure 2(a). When fouling occurs in an active segment (e.g., segment-1), the inlet and outlet ports are shifted to simulate segment movement, and the fouled segment is now isolated using the valves 1A and 1B as shown in Figure 2(b). The clean segment (e.g., segment-4) which is ready to be used can be connected after segment-3 so that the number of active segments is still three. The encrust layer essentially consists of the solute component. It is suggested that the isolated segment-1 of the PFC is cleaned by circulating warm solvent to dissolve the encrust layer. The dissolution rate is much faster as compared to the growth rate of crystals. For instance, it is reported that the dissolution rates of sodium chlorate were ∼4 times higher than the growth rates at similar driving forces/hydrodynamic conditions.^24^ Therefore, the cleaning time will be shorter as compared to the time required for the encrust layer to exceed the threshold limit. Such a cleaning strategy will also allow for having provision for recycling the API recovered by dissolving the encrust layer. These steps are repeated periodically to ensure continuous operation. However, a situation may arise when the most fouled segment is not the one where feed is introduced. In such cases, it will be required to isolate an interior segment, e.g., segment-2 in Figure 2(a). The fouled segment-2 can be isolated using the valves 2A and 2B, and the slurry from segment-1 can bypass segment-2 through the tubes shown with dashed lines in Figure 2(a). The whole process can be automated by employing valves that are controlled by a PLC (programmable logic controller). For encrustation detection, a noninvasive imaging technique can be used as outlined by Sheridan et al.^8^ In this technique, two cameras were fitted to a moving fluid oscillatory baffled crystallizer (MFOBC) at 21.5 cm apart, and images from these cameras were saved simultaneously every three seconds. These images were subsequently analyzed by visual inspection and using automated image processing methods to quantify the fouling events in terms of fouling induction time. Although the concept is presented with 3 active segments in the PFC, in principle, the number of active segments can be more than 3 if required. Finding the optimum number of active segments and the length of each segment in the PFC is discussed later in the paper. The development of the mathematical model for the crystallization process in PFC in the presence of encrustation is discussed next.

## Model Development

The plug flow crystallizer (PFC) model used in the study is based on the following assumptions:

1. The PFC is well mixed in the radial direction, and no mixing occurs in the axial direction.
2. Temperature gradients are present in the axial and radial directions across the PFC wall and encrust.
3. The cooling jacket is able to maintain the temperature of the outer wall at a constant temperature which causes the heat transfer across the PFC wall.
4. Change of the physical properties of the encrust layer, PFC wall, and solution are negligible in the operating temperature range.
5. The volumetric flow rates of feed and the product are constant.

The first assumption is applicable for an ideal plug flow reactor which can be satisfied closely in a continuous oscillatory baffled crystallizer (COBC) if it satisfies the condition that the Peclect number (Pe) is above 50.^25,26^

The PFC wall and the encrust layer consist of solid domains, and therefore, the main mode of heat transfer at these domains is conduction. As a result of the symmetric nature of the heat of conduction, the temperature gradient with respect to the angular coordinate can be neglected. A water jacket with sufficient water flow can maintain the temperature of the outer wall surface of the PFC segment at a constant temperature. The physical properties of the encrust layer, PFC wall, and solution can be assumed to be constant if the operating temperature range for the crystallization process is not large. A constant volume pump can be used to maintain the constant volumetric flow rate through the PFC.

**Figure 3:**
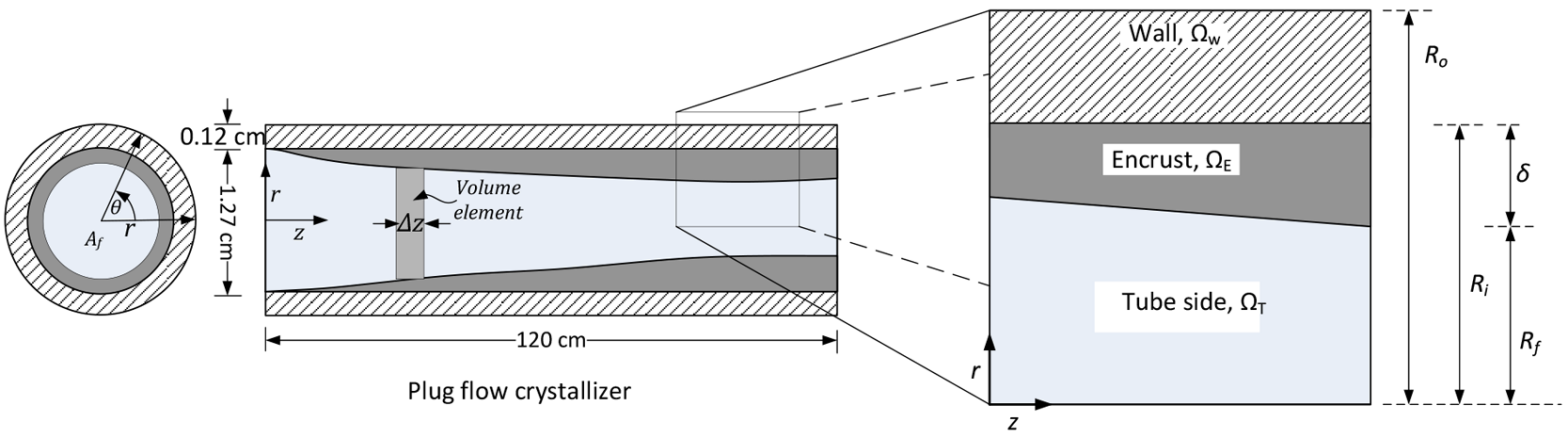
Schematic of a cross section of a PFC with encrustation. Reproduced with permission from ref (7). Copyright (2015) American Chemical Society.

A cross section of the PFC depicting various domains is shown in Figure 3. These domains are the PFC wall (Ω_W_), encrust layer (Ω_E_), and tube side (Ω_T_). The encrust formation model used in this work is based on the fouling model previously presented by Bohnet,^4^ Brahim et al.,^5^ and Majumder and Nagy.^7^

### Encrust Formation

The net amount of solute deposition on the PFC wall is calculated by considering various contributing subprocesses such as transport of the solute molecules from the bulk fluid to the heat transfer surface, attachment and deposition of the solute molecules on the surface to form the encrust layer, and removal of the encrust due to shear stress resulting from fluid flow. The final set of equations obtained to track the thickness of the encrust layer is as follows
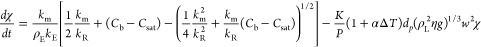
1Here, χ is the thermal resistance, *k*_m_ is the mass transfer coefficient, *K*_R_ is the surface reaction rate constant, *C*_b_ is the bulk fluid concentration, *C*_sat_ is the saturation concentration, α is the linear expansion coefficient, η is the viscosity of the liquid phase, ρ_L_ is the density of the liquid phase, *g* is the gravitational acceleration, and *w* is the fluid velocity. The rate of the change in thermal resistance described by the above equation and the rate of deposition are related as
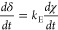
2

More details on the derivation of this equaiton can be be found in the Supporting Information.

A COBC type PFC is considered in this study where an oscillatory flow of the fluid is superimposed on the net flow that creates the turbulence required for mixing. The residence time of the slurry within the crystallizer depends on the net flow, while the heat and mass transfers due to mixing are determined by the oscillatory flow. Therefore, appropriate definition of Re should be used in heat and mass transfer calculations as shown in eq 5. During the crystallization, the encrustation also takes place causing the flow area *A*_f_(*z*) of the PFC to change with time. This in turn affects the amplitude of oscillation and local fluid velocity. Therefore, the appropriate fluid velocity *w*(*z*) in COBC for mixing can be calculated as
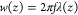
3Here, *f* is the frequency, and λ(*z*) is the amplitude of oscillation. The dependence of λ(*z*) on the flow area can be obtained from the expression of the constant volumetric flow rate along the PFC as follows
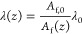
4where *A*_f,0_ and λ_0_ are the flow area and amplitude of oscillation, respectively, in a crystallizer free of encrustation. Encrustation can cause a decrease in the flow area locally and therefore an increase of the local fluid velocity *w*(*z*). The appropriate definition of Re for COBC can be expressed as^27^
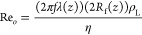
5Here, Re_*o*_ is the oscillatory Reynolds number which is calculated from the oscillatory velocity responsible for mixing. The frequency and amplitude of oscillation used in this study are *f* = 2 s^–1^ and λ = 0.04 m, respectively.

### Energy Balance

#### Tube Wall

The primary mode of heat transfer in the tube wall domain Ω_W_ = {(*r*, *z*) : *r* ∈ [*R*_i_, *R*_*o*_], *z* ∈ [0, *Z*]} is conduction. The standard heat conduction equation assuming symmetry with respect to the angular coordinate, θ, without any source term can be written as
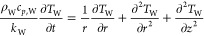
6Here, ρ_W_ is the wall density, *c*_*p*,W_ is the wall specific heat capacity, *T*_W_(*z*, *r*) is the wall temperature, and *k*_W_ is the wall thermal conductivity.

#### Encrust Layer

The heat transfer in the encrust domain Ω_E_ = {(*r*, *z*) : *r* ∈ [*R*_f_(*z*), *R*_i_], *z* ∈ [0, *Z*]} can be modeled similarly to the tube wall as
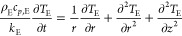
7where ρ_E_ is the encrust density, *c*_*p*,E_ is the encrust specific heat capacity, *T*_E_(*z*, *r*) is the encrust temperature, and *k*_E_ is the encrust thermal conductivity. The boundary of the encrust domain Ω_E_ changes with time as the encrust layer grows or dissolves. Therefore, it possesses a moving boundary problem. We further note from Figure 3 that the flow radius *R*_f_(*z*) is related to encrust thickness δ(*z*) as follows
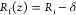
8In order to tackle this problem while solving eq 7, it is convenient to reformulate the equation in terms of dimensionless radial coordinate *r̃* defined as follows^28^
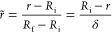
9Therefore, we have
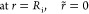
10
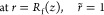
11It is to be noted that in the newly defined coordinate system, the base is at *R*_i_, and the direction is reversed. With this definition of the coordinate *r̃*, eq 7 can be expressed as follows
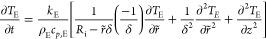
12

#### Tube Side

The tube side domain Ω_E_ = {(*r*, *z*) : *r* ∈ [0, *R*_f_(*z*)], *z* ∈ [0, *Z*]} consists of a slurry, and therefore, the main modes of heat transfer are the convection and conduction along the axial direction and the overall heat transfer through the interface between the tube side and encrust layer. Since the encrust thickness changes with time, it will also result in changes in the flow area of the tube. This change in the flow area needs to be accounted for while carrying out the heat balance. The final form of the heat balance can be written as^7^
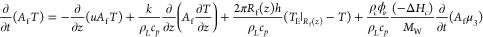
13Here, *A*_f_ = *πR*_f_^2^ is the flow area, *u* is the mean flow velocity, *k* is the thermal conductivity at the tube side, *h* is the overall heat transfer coefficient, Δ*H*_*c*_ is the heat of crystallization, ϕ_*v*_ is the volume shape factor for the crystals, and *M*_W_ is the molar mass of the crystal.

#### Boundary Conditions

The PDEs describing the energy balance need to be supplemented with the appropriate boundary conditions. At the interface between the tube wall Ω_W_ and encrust layer Ω_E_, the continuity of heat flux and temperature results in the following boundary conditions
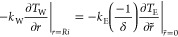
14
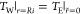
15Similarly, the continuity of the heat flux at the interface between the encrust Ω_E_ and tube side Ω_T_ and the known inlet temperature of the feed, *T*_in_, lead to the following boundary conditions
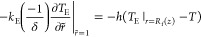
16
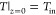
17

### Population Balance Model for Crystallization

**Figure 4:**
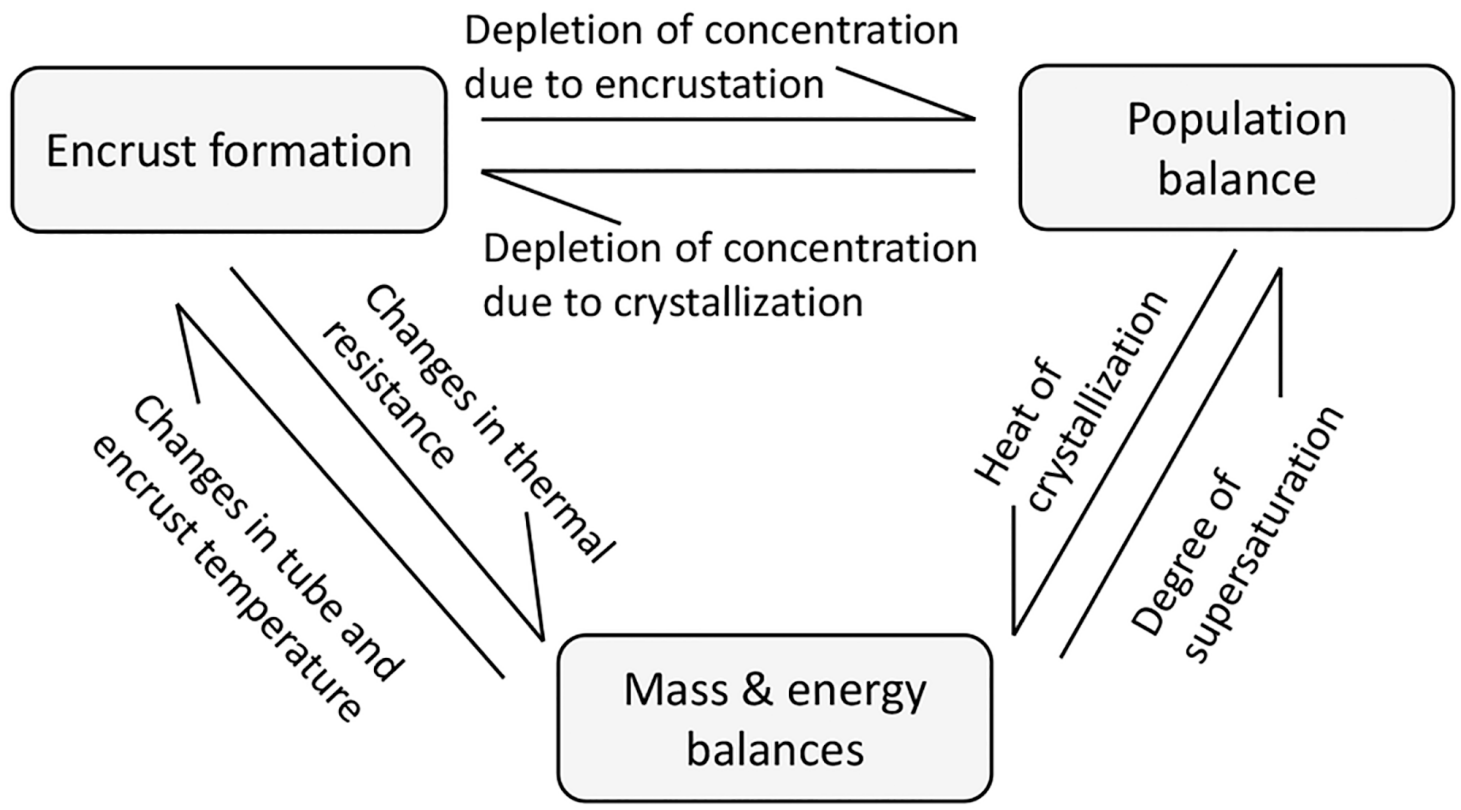
Schematic showing the interactions of the model components required for the simulation of the crystallization process in the presence of fouling. Due to the coupling of the governing equations, they need to be solved simultaneously. Adapted with permission from ref (7). Copyright (2015) American Chemical Society.

The evolution of the crystal size distribution within the PFC can be modeled using population balance equations (PBEs). Since the crystal growth and nucleation depend on the supersaturation, temperature, and amount of crystals present, the PBE is strongly coupled with encrust formation, mass, and energy balance equations. Therefore, the governing PBEs need to be solved simultaneously. The interactions of the various model equations are depicted in the schematic diagram shown in Figure 4

In order to calculate the encrust thickness in eq 2, the concentration inside the PFC is required. Thus, the governing equations for encrustation have to be coupled with the crystallization model which is represented by population balance and mass balance equations. The change in the flow area in the tube side due to encrust formation has to be taken into account while deriving these balance equations. The PBE describing crystallization with one size coordinate and one spatial coordinate can be written as
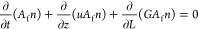
18
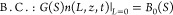
19
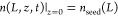
20where *n* is the CSD, *G* is the crystal growth rate, *L* is the crystal size, *n*_seed_ is the seed distribution at the feed, *B*_0_ is the nucleation rate, and *S* is the supersaturation. The kinetic equations for growth and nucleation are presented in the simulation study section. Note that the cross-sectional area of the PFC, which is varying with time due to encrustation, appears in the PBE. The detailed derivation of this equation can be found in the Supporting Information of Majumder and Nagy.^7^ Now, the mass balance equation that takes into account the change in solution concentration, *C*, due to crystallization and encrustation can be written as^7^
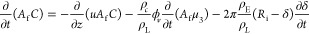
21where μ_3_ is the third moment of CSD. The encrust density (ρ_E_) can be calculated by combining the crystal density (ρ_c_), liquid phase density (ρ_L_), and encrust void fraction (ϵ) as follows
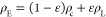
22

## Numerical Method Used for Solving the Governing Equations

The solution of the coupled system of governing equations discussed in the previous section requires the use of numerical methods in most practical cases. Numerical solution of the PBEs can be challenging as they are hyperbolic PDEs where discontinuities or sharp changes in solution domain may arise. Using higher order spatial discretization to capture these features can result in numerical oscillations, while first order discretization can result in dispersions. In this work, a widely used high resolution finite volume method is used to solve the governing hyperbolic PBEs. This particular method uses the van Leer flux limiter^29−31^ combined with the finite volume method to capture the discontinuity or sharp changes of the variables with respect to the spatial domain without significant dispersion. The details of the method are not discussed here for brevity and can be found elsewhere.^7,29,30,32^ The other governing PDEs can be discretized using the finite difference technique to obtain corresponding ODEs. It has been found that the coupled system of governing equations forms a stiff system of ODEs after spatial discretization. Therefore, all the resulting ODEs are then solved using the solver ODE15s which is designed to solve stiff differential equations available in MATLAB.

## Case Study Demonstrating Effectiveness of Cleaning Events

A simulation study is carried out to demonstrate the effectiveness of the proposed SM-PFC configuration to tackle the encrustation problem. The case study considered is taken from Majumder and Nagy^7^ where cooling crystallization of the potash alum-water system is investigated. The PFC studied in this simulation consists of five segments with four segments in active use at any given time. The length of each segment is 1.80 m. Therefore, the total length of the PFC is 7.20 m. The PFC is made of pyrex glass with an internal diameter of 1.27 cm and wall thickness of 0.12 cm. The saturated solution at 40 °C is fed to the crystallizer, and the seed distribution in the feed, *n*_seed_ # m^–1^ m^–3^, is given as
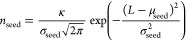
23where κ = 1 × 10^10^ is a scaling factor corresponding to 1.88% of seed mass, σ_seed_ = 15 × 10^–6^ m is the standard deviation, and μ_seed_ = 54 × 10^–6^ m is the mean size. The feed flow rate to the PFC is 100 mL min^–1^. A cooling jacket is used to maintain the outer wall temperature at 30 °C so that the feed slurry is cooled as it passes through the PFC due to the heat transfer through the PFC wall. As a result, supersaturation is generated in the PFC, and crystallization takes place. Nucleation and growth kinetics for the potash alum-water system are taken from Shoji et al.^33^ The nucleation rate is contributed by both the primary and secondary nucleation rates as follows
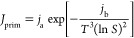
24
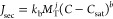
25where *j*_a_, *j*_b_, and *k*_b_ are nucleation parameters, *M*_T_ is the magma density, and *S* is the supersaturation ratio defined as
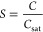
26Therefore, the total nucleation rate is the sum of the primary and secondary nucleation rates. The crystal growth rate, *G*, can be expressed as a function of the relative supersaturation, temperature, and crystal size^33^ using an empirical expression as follows
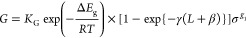
27where *K*_G_ is the growth rate constant, Δ*E*_g_ is the activation energy for growth, γ, β, and *g*_1_ are growth parameters, and σ is the relative supersaturation calculated as
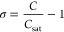
28The parameters for growth and nucleation kinetics are given in Table 1.^7,33^

**Table 1:** Growth and Nucleation Kinetic Parameters Used in the Simulation^a^

parameter	value	units
*j*_a_	1.70 × 10^8^	# m^–3^ s^–1^
*j*_b_	5.64 × 10^6^	K^3^
*k*_b_	3.14 × 10^7^	# m^–3^ s^–1^
*j*	1	-
*b*	1.32	-
*K*_G0_	2.05 × 10^5^	m s^–1^
γ	7.18 × 10^2^	-
β	6.10 × 10^–5^	M
Δ*E*_g_	5.77 × 10^4^	J mol^–1^
*g*_1_	1.42	-

a Reproduced with permission from ref (7). Copyright (2015) American Chemical Society.

The parameters for encrust formation rate are taken from a previous study by Majumder and Nagy^7^ and are listed in Table 2. Since the encrustation parameters for potash-alum are not available, the parameters listed in Table 2 are the experimentally determined parameters for another inorganic salt CaSO_4_ except for higher values for the reaction rate constant to emphasize encrust formation. Moreover, it is assumed that the heat of crystallization is negligible compared to the other contributing terms in the energy balance equation. The simulations were performed on a Linux workstation (Intel Xeon 6230 2.1 GHz (20 Core), RAM: 128GB RAM) using MATLAB 2020b. The number of grid points used was 20 along axial, radial, and size axes.

**Table 2:** Parameters Used in Simulation for the Wall, Encrust, and Tube Side Domains^a^

domain	parameter	value	units	reference
wall	ρ_*W*_	2230	kg m^–3^	pyrex glass
	*c*_*p*,W_	753	J kg^–1^ K^–1^	pyrex glass
	*k*_W_	1.005	W m^–1^ K^–1^	pyrex glass
encrust	ρ_E_	1750	kg m^–3^	potash alum
	*c*_*p*,E_	870	J kg^–1^ K^–1^	sodium chloride
	*k*_E_	1.11	W m^–1^ K^–1^	CaSO_4_^4^
	*K*_R0_	7.07 × 10^6^	m^4^ kg^–1^ s^–1^	-
	*d*_*p*_	36	μm	Brahim et al.^5^
	*D*	1.57 × 10^–9^	m^2^/s	Bohnet^4^
	*E*	37143	J mol^–1^	Brahim et al.^5^
	α	1 × 10^–6^	K^–1^	Bohnet^4^
	η	600 × 10^–6^	Pas	Bohnet^4^
	ϵ	0.2	-	Bohnet^4^
tube	ρ_L_	1080	kg m^–3^	water
	*c*_*p*_	4185.5	J kg^–1^ K^–1^	water
	*k*	0.58	W m^–1^ K^–1^	water
	*h*	1000	W m^–2^ K^–1^	shell-and-tube exchanger

a Adapted with permission from ref (7). Copyright (2015) American Chemical Society.

**Figure 5:**
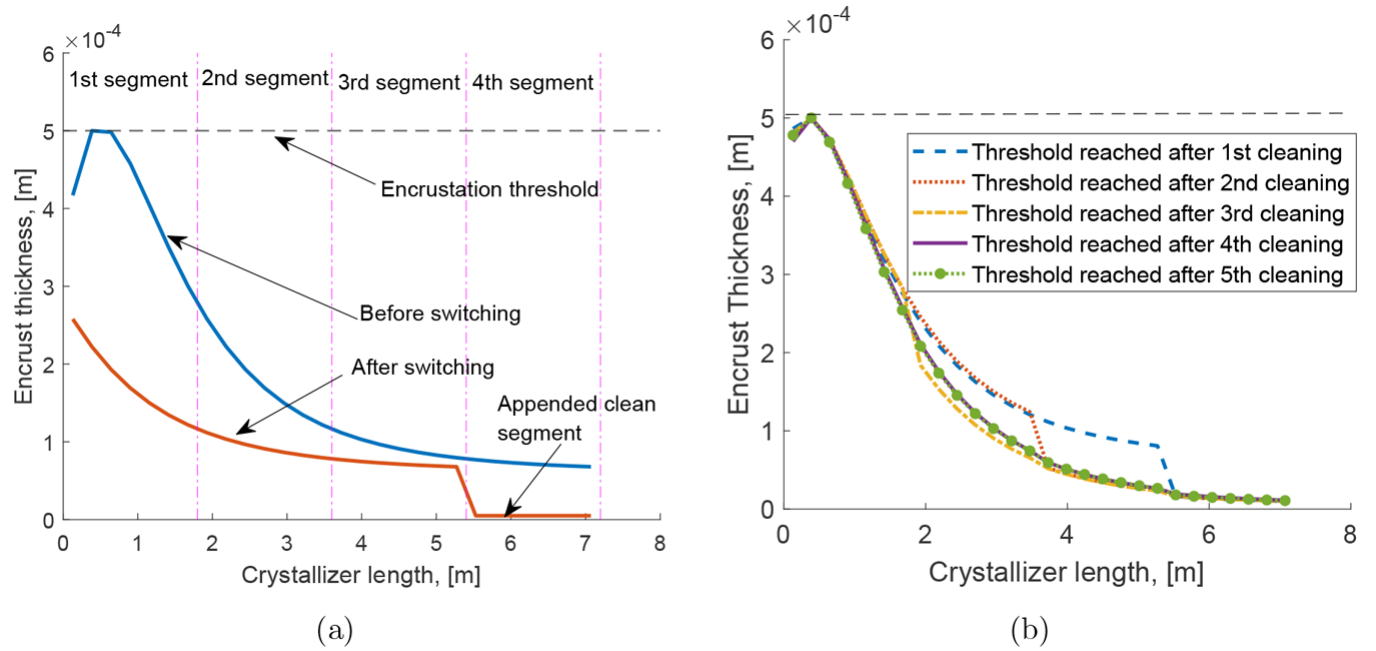
(a) Encrustation profiles in a PFC with four active segments found before and after the encrust control strategy is first implemented. (b) Encrustation profiles immediately preceding cleaning events, i.e., when cleaning criterion met.

The SM-PFC system is then simulated with a threshold cleaning criterion corresponding to the encrust thickness of 0.5 mm. Although this threshold encrust thickness is arbitrary at this stage, this can be chosen by the user based on the system being investigated so that the encrustation does not affect the crystallization process significantly. In Figure 5, the left plot shows the encrustation profile at the moment the cleaning criterion (encrust thickness of 0.5 mm or greater) is met which is about half an hour after the crystallization has started. In accordance with the SM-PFC cleaning procedure, the most fouled segment, which is the first segment in this case, is isolated from the PFC for cleaning with the remainder of the crystallizer left in operation. A clean segment is then added to the end to replace the removed segment, thereby retaining the total length of the crystallizer. The corresponding encrust profile immediately following a cleaning procedure is also shown in Figure 5(a). Once the first segment is removed and the clean segment is appended, this action mimics the movement of the encrust thickness profile to the left. Therefore, the second segment before the cleaning event works as the first segment where feed will be introduced after the event. A discontinuity is observed near the end of the encrust thickness profile. This signifies a clean segment which has replaced the one removed.

**Figure 6:**
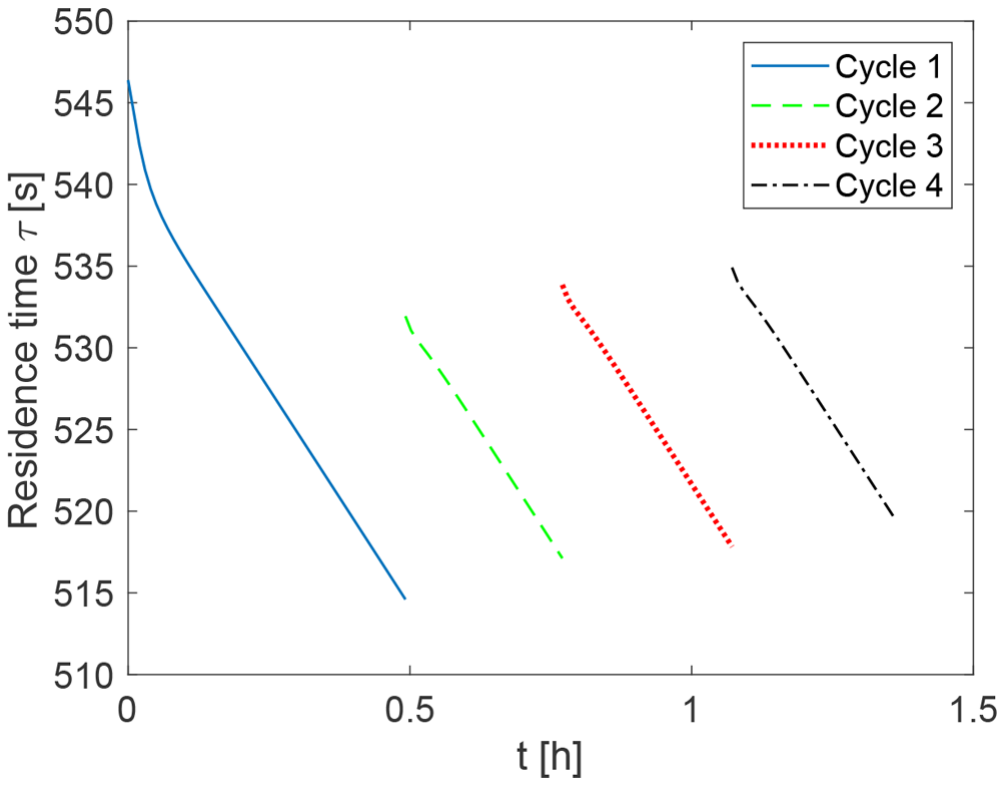
Evolution of the residence time due to encrustation and its removal through cleaning. The discontinuities and sudden jumps in the curve denote the cleaning events.

By performing several more cleaning events and once again plotting the encrust thickness profiles, Figure 5(b) is obtained. These encrust thickness profiles are taken at the instant preceding the cleaning events, i.e., the instant when the encrust thickness reaches the threshold value following a cleaning event. For instance, the profile ‘Threshold reached after first cleaning’ is obtained after continuing the simulation shown in Figure 5(a) until the cleaning criterion is met once more. Once the cleaning procedure has occurred and operation continues until the criterion is met once more, we obtain the profile ‘Threshold reached after second cleaning’ and so on in Figure 5(b). The step changes, which are most notable in the aforementioned two profiles (at 5.5 and 3.5 m, respectively), are a result of the segment switching process. The duration of the crystallization operation between the cleaning events can be found in Figure 6 which clearly shows that the first cycle is longer than the subsequent cycles.

By superimposing the encrustation profiles, we observe that this system appears to be tending toward a cyclic steady state (with respect to the encrust thickness profile). This steady state profile appears after performing as many cleaning procedures as there are segments in the crystallizer. This can also be viewed as a complete regeneration of the crystallizer. In other words, we have a four-segment crystallizer which has had four segments removed and replaced. It has been found that the encrustation profiles immediately before the subsequent cleaning events are very similar as may be expected of a periodic steady state profile. Two such profiles are shown in Figure 5(b) which indicates the profiles after the 4^*th*^ and 5^*th*^ cleaning events are visually almost identical.

We can also examine how the buildup of encrustation (and thereby the reduction of flow area or reactor volume) affects the residence time of the reactor. This is shown in the following plot in Figure 6. The plot has four lines: the first showing how the residence time of an initially clean reactor evolves through time. This first cycle starts with a higher value of residence time than the following cycles as there is no existing encrust layer in the crystallizer at the start of the crystallization process. This means the maximum flow area is available; hence, the flow velocity is as low as possible giving the highest possible residence time. As the encrust layer builds, the flow area is reduced, and the flow velocity must increase (as the feed rate is held constant). Therefore, the development of the encrust layer reduces the residence time throughout the operation. There is a discontinuity between the first cycle profile and the second profile. This discontinuity arises from the cleaning event: we consider that the most heavily encrusted segment is removed instantaneously, and a clean segment replaces it, also instantaneously. This instantaneous increase in the reactor volume or average flow area (through replacing a fouled segment with a clean one) results in an instantaneous increase in the residence time. The operation then continues, with the encrust layer building across the reactor, reducing the residence time, until the cleaning criterion is met. The cleaning event occurs once more, and the cycle repeats. Thus, we reach a cyclic steady state where the residence time reduces from 535 s to 520 s, and the time between the cleaning events at steady state is roughly 20 min.

## Development of a Regime Map for Crystallizer Design

The aim of this section is to demonstrate how a regime map can be developed with the knowledge of the crystallization system (e.g., nucleation, growth, and encrustation kinetics) being investigated. Using this regime map, users in principle would be able to design crystallization processes (e.g., temperature profile, residence time, seed mass, number, and length of crystallizer segments) which will allow them to achieve the required objectives (e.g., productivity, mean crystal size) in the presence of fouling. Development of such a regime map is discussed in the subsequent sections.

### Selection of Crystallization Kinetic Parameters for the Regime Map

The nucleation and growth kinetics are the two phenomena which are generally of the greatest interest in crystallization processes. However, for systems such as the PFC where clogging or other operational issues can occur due to fouling, we are also interested in how quickly the encrustation layer develops. This gives three distinct phenomena: nucleation, growth, and encrustation which are of special interest for investigating crystallization processes with fouling. However, if the aggregation or the breakage phenomena dominate a certain crystallization process such as in spherical crystallization process,^34,35^ then these phenomena have to be considered as well. Nevertheless, aggregation and breakage phenomena are not considered in this study. Maldonado et al.^36^ has done an extensive literature review and presented sampling distribution of growth and nucleation kinetic parameters. Since in this study the primary objective is to demonstrate that the proposed design of the PFC system can handle the occurrence of heavy fouling during crystallization, we select the kinetic parameters for simulation studies such that the crystallization system would represent a high-encrustation moderate-growth moderate-nucleation system. Crystal growth rate expression used in this regime map development is one of the most widely used expressions in the literature^36^
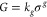
29Hence, we have only two growth parameters: the growth rate constant *k*_*g*_ and the growth exponent *g*. However, the expressions for primary and secondary nucleations remain the same as in the previous section. The kinetic parameters which have been used to define a high-encrustation moderate-growth moderate-nucleation system are summarized in the following Table 3.

**Table 3:** Kinetic Parameters Used to Define a High-Encrustation Moderate-Growth Moderate-Nucleation Crystallization System That Has Been Used for the Development of the Regime Map

parameter	value	units
*j*_a_	10^8^	# m^–3^ s^–1^
*j*_b_	10^6^	K^3^
*k*_b_	5 × 10^15^	# m^–3^ s^–1^
*b*	1.0	-
*K*_g_	10 × 10^–6.5^	m s^–1^
*K*_R0_	7.07 × 10^6^	m^4^ kg^–1^ s^–1^
*g*	1.0	-

Now a regime map can be developed for this crystallization system by performing optimization of the process. This regime map will contain information such as what objectives in terms of productivity and mean crystal size are achievable when using a PFC system with certain feed rate, seed mass, and temperature profiles. The formulation of the optimization problem is discussed in the next section.

### Formulation of the Optimization Problem to Develop the Regime Map

In a crystallization process, two of the most important performance criteria are the product quality and yield. Poor product quality may result in, for example, poor bioavailability, while poor yield indicates an incomplete reaction, receiving lower product quantity. We wish to maximize the volume mean crystal size and maximize the productivity. We would also like to maximize duration between the two cleaning events, i.e., minimize the frequency of the cleaning process. Therefore, the results generated in this section were the product of a multiobjective optimization rather than a single objective optimization. It is to be noted that in a multiobjective optimization, there is no longer an objectively optimal solution. There will exist a trade-off between competing objectives. This trade-off can often be defined qualitatively and quantitatively and takes the name Pareto front or Pareto surface. The Pareto front therefore represents the nondominated solutions of the optimization. From these solutions, an improvement in any given objective cannot be achieved without sacrificing improvement in at least one other objective. With three objectives, this optimization can be represented in three dimensions, with the Pareto front taking the form of a Pareto surface. The decision variables considered are pertinent to the design of the crystallization process in an SM-PFC system. These are the coolant temperature profile along the PFC (this is taken to be the same as the outer wall temperature of the PFC based on the assumption that the cooling jacket is well-mixed due to the high flow rate of the coolant^25^), feed rate, seeding rate to the crystallizer, number of segments, and length of each segment of the PFC. The temperature profile is approximated by a number of the equally spaced temperatures along the outer wall depending on the spatial discretization used in the numerical method. Mathematically the formulation of the optimization problem can be summarized as
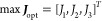
30
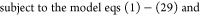
31
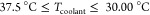
32
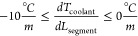
33
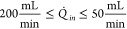
34
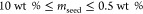
35
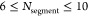
36
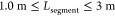
37Here, *T*_coolant_ is the temperature of the coolant in the cooling jacket of the crystallizer which is the same as the outer wall temperature profile of the PFC,  is the volumetric flow rate of the feed, *m*_seed_ is the amount of seed added, *N*_segment_ is the number of segments in the crystallizer, and *L*_segment_ is the length of each crystallizer segment. The limits for the decision variables are obtained by analyzing some of the experimental results published in literature.^1,37^ The three objective functions are the volume mean crystal size (defined as the ratio of the fourth moment and the third moment of the CSD)
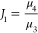
38mean productivity
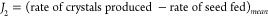
39and the time between the two subsequent cleaning events
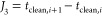
40Here, *t*_clean,*i*_ is the time at the *i*^*th*^ cleaning event. A maximum allowed coolant temperature gradient along the PFC is taken ,10 [°C/m], to avoid unrealistic temperature profiles suggested by the optimizer. Moreover, only cooling crystallization is explored, and therefore, heating is not considered. The optimization problem has been solved numerically using the Global Optimization Toolbox available in MATLAB. Several algorithms have been tested which are simulated annealing, particle swarm, pattern search, and genetic algorithm (GA). Among these algorithms, the GA has been found to provide the best balance of speed and optimization capability. Therefore, the results obtained with the GA are presented here. It is to be noted that in this optimization problem, the number and the lengths of each segment of the PFC segments are also the decision variables. This essentially means that the total length of the PFC may vary, and therefore, the number of axial discretization points used will also need to be varied in order to have the same level of resolution along the axial direction while solving the model equations numerically. This varying number of axial nodes results in a varying number of decision variables (e.g., coolant temperature at these nodes). Implementation of a varying number of decision variables in the GA within the MATLAB Global Optimization Toolbox is not trivial once the optimization has started. In order to overcome this issue, we have run the optimization problem for different combinations of the segment lengths (range considered 1.0 m–3.0 m) and number of segments (range considered 6–10).

The length and the number of segments used in this optimization study are summarized in Table 4. An empty element is shown for the crystallizer having 10 segments with each segment being 3 m long. This run is considered impractical due to the length of time required to simulate Run 8 (approximately 10 days) and therefore not investigated.

**Table 4:** Optimization Matrix for the Optimizations Performed Showing the Dimensions of Each Crystallizer Simulated and Their Respective Numbering

		no. of segments		
		6	8	10
segment length [m]	1	Run 1	Run 2	Run 3
	2	Run 4	Run 5	Run 6
	3	Run 7	Run 8	-

### Optmization Results for the Development of the Regime Map

As discussed in the previous section, optimization of the crystallization process is carried out for each of the PFC configurations (with varying number and length of segments) shown in Table 4. First, a representative PFC configuration is chosen, and the optimization results in terms of the obtained objective functions and decision variables are discussed in detail. Subsequently, the results for all the PFC configurations are collated to generate the regime map.

**Figure 7:**
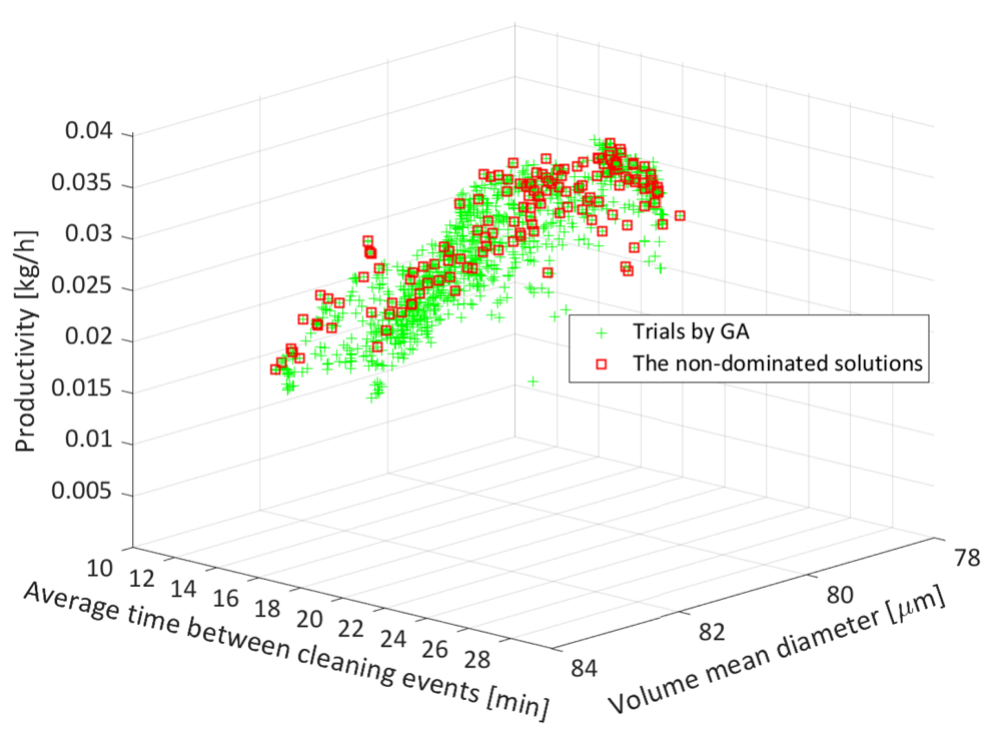
Optimization results for the PFC with 10 segments each having a length of 1 m. Optimization is performed with respect to three objectives, which are shown on the three axes. The Pareto surface consisting of nondominated solutions is highlighted using the square markers.

The optimization results for the PFC configuration with 10 segments each having a length of 1 m, i.e., Run 3 from Table 4, are presented as the representative case study. The simulation parameters used are the number of grid points for space *N*_*z*_ = 30, number of grid points for crystal size *N*_*L*_ = 22, and number of grid points in radial direction *N*_*r*_ = 8 both in encrust and wall domains. The corresponding objective functions are shown in Figure 7. Each point (represented by a green marker) within the objective function space represents a trial by the GA involving evaluation of the three objective functions corresponding to a combination of decision variables. In the plot are shown 3636 data points, each representing a candidate of some generation within the algorithm. This plot is therefore the result of simulating the process 3636 times, with various combinations of decision variables in an attempt to find the optimal solutions with respect to the three objectives. Due to the way the GA works, some combinations of the decision variables and therefore the objective functions can be repeated. Only a subset of these trial or candidate points shown in Figure 7 will represent the optimal points. These points are highlighted by red markers and known as the nondominated solutions. The Pareto surface is created by the collation of these nondominated solutions. As 3D plots can be difficult to interpret in 2D form, the following summary gives the range for each objective for the nondominated optimal points. The volume mean diameter ranges from 81 to 84 μm, productivity ranges from 0.02–0.04 kg/h, and the average time between the two cleaning events ranges from 16–27 min. It is to be noted that the high frequency of cleaning events reflects the large value of the encrustation rate that is used in these simulations. The high encrustation rate will also result in lower productivity as some solute is lost due to formation of the encrust layer.

**Figure 8:**
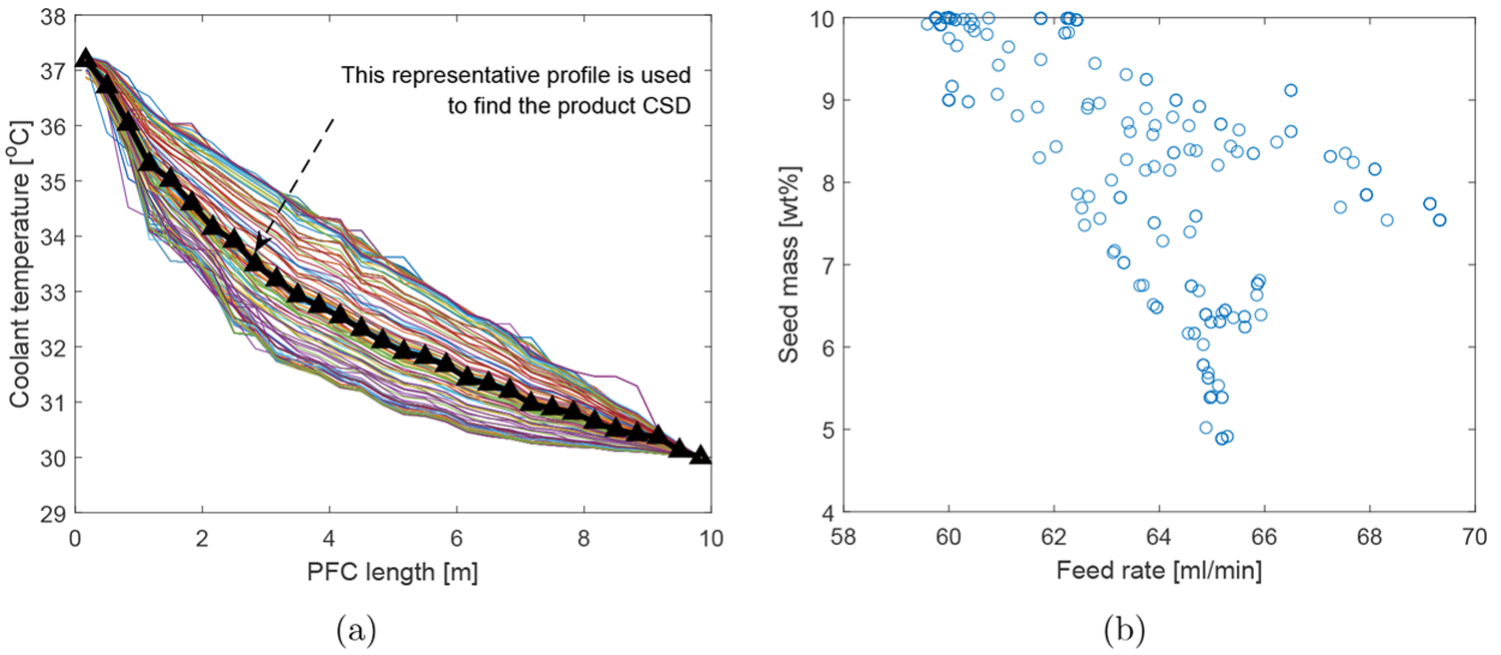
Decision variables (temperature profiles, feed rate, and seed mass) corresponding to the Pareto optimal points shown in Figure 7.

The decision variables, i.e., coolant temperature profile, feed rate, and seed mass, corresponding to the Pareto optimal points or the nondominated points in Figure 7 are shown in Figure 8. It can be seen that the optimizer has explored a wide range of temperature profiles starting from exponentially decaying temperature profiles of various degrees to a linear profile. An exponentially decaying cooling profile which resembles natural cooling would mean that the driving force for crystallization, i.e., supersaturation, will be high in the segments close to the inlet. This can lead to a large amount of secondary nucleation for a highly nucleating system.^38^ The controlled cooling profile which involves slow cooling at the beginning and fast cooling toward the end is more suitable for such systems. However, in this study, a moderately nucleating system is investigated. Therefore, a controlled cooling profile is not considered by the optimizer as it would not lead to an optimal crystallization operation. The other two decision variables, feed rate and seed mass, are also shown in Figure 8 that corresponds to the Pareto optimal points. It can be seen that the seed mass is well spread between 5 and 10 wt %, although a good number of optimal points lie at the upper limit (i.e., 10 wt % seed mass). A higher seed mass provides a larger surface area for the crystal seeds to grow and is associated with higher productivity. In contrast, the optimal values for the feed rate are biased toward its lower limit (range considered 50–200 mL/min). This can be explained as a lower feed flow rate will increase the residence time of the seeds in the PFC and therefore will be favorable for crystal growth.

### Collation of the Optimization Results to Generate the Regime Map

**Figure 9:**
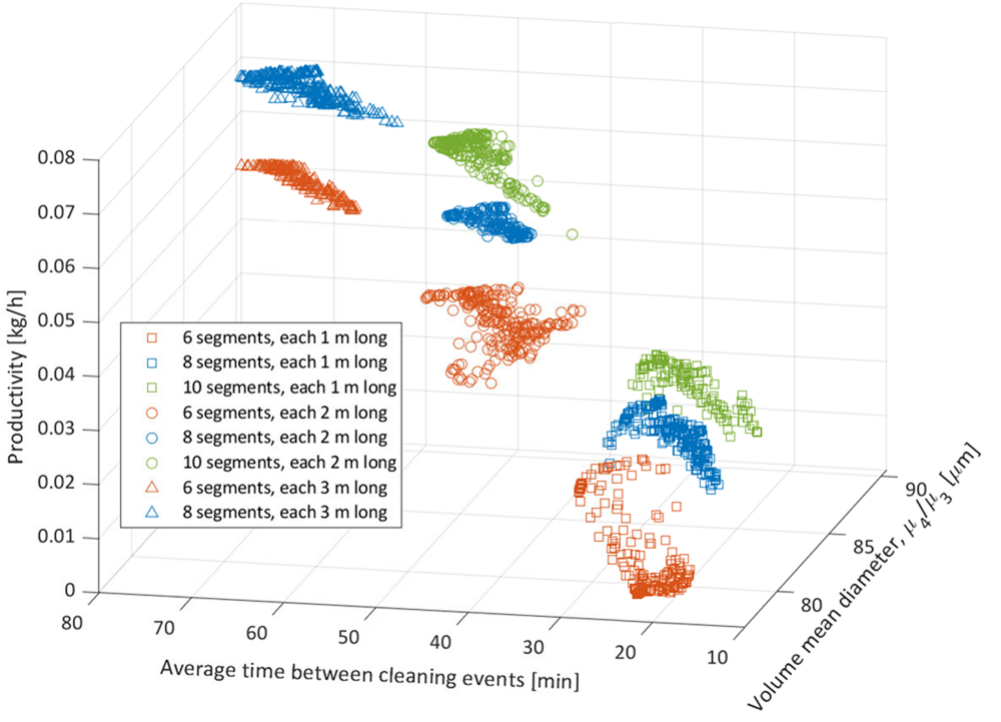
Regime map developed for a high-encrustation moderate-growth moderate-nucleation crystallization system by collating the optimization data for all simulated crystallizers. Crystallizers with an equal number of segments are displayed in the same color. Crystallizers with the same length of segment are displayed with the same marker.

The optimization results discussed in the previous sections correspond to a single PFC configuration which is ‘Run 3’ from Table 4. Results obtained for all these crystallizer configurations can be collated to generate the regime map for a high-encrustation moderate-growth moderate-nucleation crystallization system as shown in Figure 9.

It is interesting to discuss the direct application of the information shown in Figure 9. For a user who wishes to use the SM-PFC, who knows their crystallization regime and desired productivity and/or product crystal size, these plots can quickly suggest the number and length of segments required for that PFC. Each cluster in the regime map corresponds to the optimal points for a crystallizer configuration with a certain number and length of segments. A single data point within the cluster corresponds to a set of decision variables within the design space for that PFC configuration leading to the achievement of the objectives represented by the point. Therefore, a user could either identify which range of process outcomes they could achieve with a given crystallizer or which crystallizer to select in order to achieve a predetermined outcome in terms of the performance index such as mean crystal size and productivity. If required, the combination of the size and the number of segments considered in Table 4 can be increased to obtain more clusters such as the ones shown in Figure 9. This will allow more flexibility in finding the optimal PFC configurations.

### Effect of Crystallizer Length on Objectives

The residence time of the PFC can be increased by increasing the length of the PFC. It is interesting to see that the effect of a larger residence time on some of the objective functions can be different depending on how the PFC length was increased. For instance, one can increase the length of the PFC by increasing the number of segments without changing the segment length or increasing the segment length without changing the number of segments. In Table 5 it is shown how each method of increasing residence time impacts the optimization objectives.

**Table 5:** Changes to Optimization Objectives with Respect to the Crystallizer Length Obtained by Increasing Segment Length or Increasing Number of Segments^a^

	change in no. of segments	change in length of segments
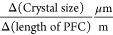	0.54	0.51
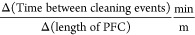	0.51	2.79
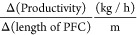	4.17 × 10^–3^	2.36 × 10^–3^

a This data is calculated for the high-encrust moderate-growth moderate-nucleation regime.

It can be seen that the rate of increase in the mean crystal size with respect to the PFC length is almost the same in both approaches. However, the most notable difference is observed for the time interval between the cleaning events which scales far more strongly when the length of segments increases as compared to when the number of segments increases. This effect arises due to the portion of the encrust removed in a single cleaning step. If a crystallizer is constructed of a few, long segments, it may take some time for that volume of encrust removed to be redeposited. Conversely, a crystallizer constructed of many short segments would remove only a small fraction of the encrust; hence, the time for this same amount to be redeposited is lower. For this reason, the increase in the time between cleaning events is larger when that increase is affected by the lengthening of segments rather than the increase in the number of segments.

Another notable effect is that of the change in productivity with length allocation. When the residence time is increased by increasing the number of segments, a greater increase in productivity is observed than when the same increase in the residence time is affected by the lengthening of segments. This can be explained by the fact that in a cleaning event, a fouled segment is replaced by a clean one containing only a saturated solution at the end of the PFC. Therefore, no product crystals will be obtained immediately after the cleaning event for a duration corresponding to the residence time of the clean segment. As a result, when a longer segment is replaced, this period of operation that does not produce product crystals increases affecting the productivity adversely. Moreover, a particularly large, clean segment presents ample opportunity for the deposition of the solute; this unfavorably affects the balance between useful solute deposition and unwanted solute deposition on the crystallizer surface. Users must therefore balance their willingness or ability to frequently clean segments with their desire for productivity.

It will also be interesting to investigate whether the SM-PFC configuration offers any advantage over simpler configurations where there are only two PFC segments and they are swapped in and out for periodic cleaning. We consider two different PFC configurations of the same total active length of 10 m with one PFC configuration having only one active segment (length 10 m) while the other having ten active segments (length 1 m each). Similar conclusions have been found as discussed above, i.e., the PFC with a segment length of 10 m provides a similar mean crystal size and longer duration between the cleaning events; however, the productivity is lower as compared to the PFC with a segment length of 1 m. More detailed discussion can be found in the Supporting Information.

### Variation in Product Qualities in Response to Cleaning Events

The cleaning strategy investigated in the operation of the SM-PFC involves the removal of the most heavily encrusted segment and addition of a clean segment to the crystallizer. This procedure is enacted to avoid clogging or other flow issues. It is worth investigating if such a procedure will have a significant effect on the product crystal qualities such as volume mean size. When a cleaning event occurs, the crystals approaching the end of the final segment (i.e, once it is near the outlet of the reactor) will have one more segment to flow through as a clean segment has been added onto the end of the crystallizer. This results in an increase in the residence time for those crystals. Such an increase in the residence time is also applicable to all the crystals that are present in segments succeeding the removed segment at the time of the cleaning event. For instance, if the first segment is removed, all crystals present at the moment of the cleaning event will have increased residence time; however, if an intermediate segment is removed, only crystals in those segments following the removed segment will have increased residence time. This may result in a slight increase in the mean crystal size of the product crystals for a brief period of time. Moreover, when a clean segment is added, if it is filled with a saturated solution, this will show up as a discontinuity in the CSD evolution profile as can be seen in the case study discussed below.

**Figure 10:**
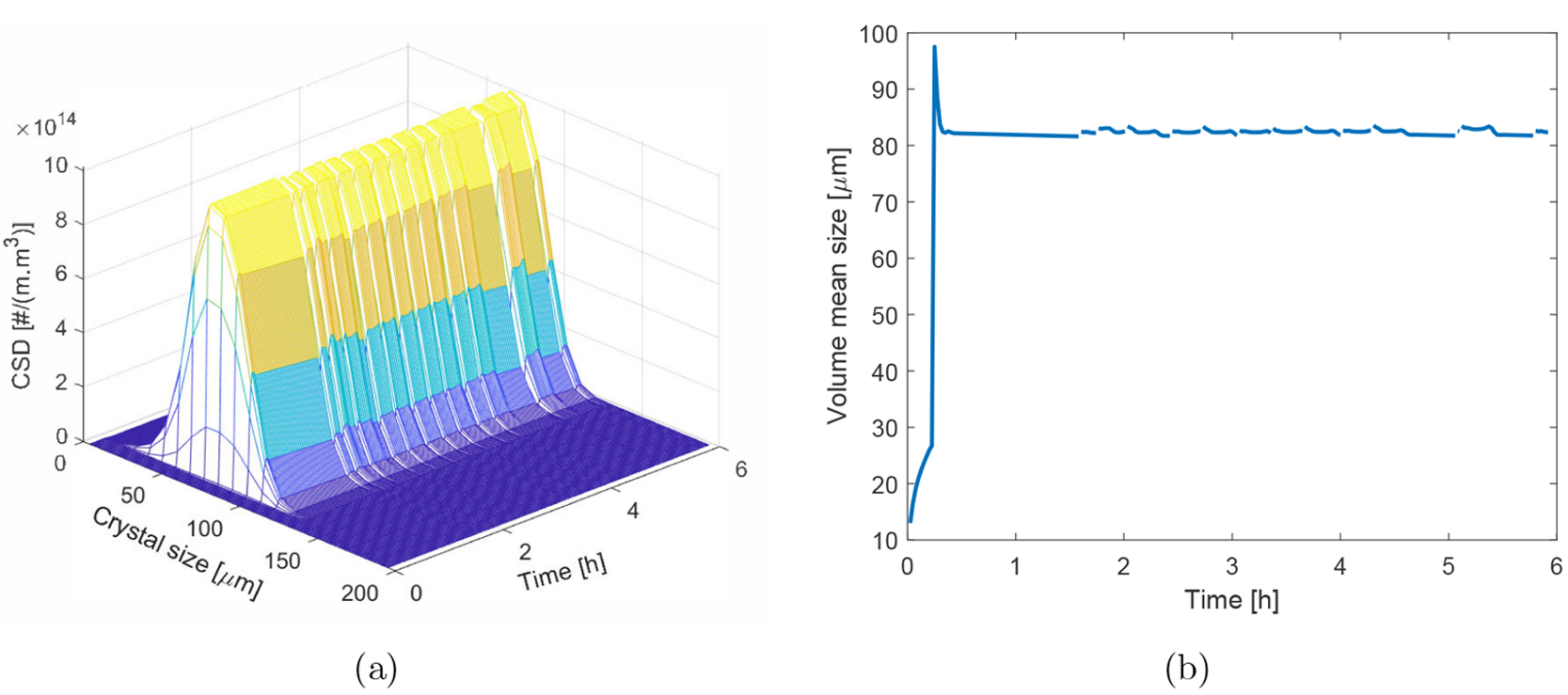
(a) Variation in the CSD and (b) variation in the volume mean diameter with the production time from startup, spanning various cleaning cycles. Results shown are for a ten-segment crystallizer; segments are each 1 m in length.

We take the case study of a PFC with ten 1 m long segments with a representative optimal temperature profile as shown in Figure 8(a). The other decision variables corresponding to the temperature profile are the feed rate 63.9 mL/min and seed mass of 8.03 wt %. The evolution of the product CSD and the volume mean diameter of the product crystals are shown in Figure 10. It can be seen in the product CSD that no crystals are present in the product immediately following startup, as seed crystals have not yet reached the crystallizer outlet. After crystals reach the outlet of the reactor, visually there is little variation in the product CSD except for a few discontinuities in the time domain that indicate the cleaning events when a new segment containing a saturated solution is added at the end of the crystallizer. It will be more evident when we look at the evolution of the volume mean size of the product crystals in Figure 10(b). A spike in volume mean size is observed near the startup of the process which can be attributed to the fact that the PFC contains a higher solute concentration at the start of the process as compared to the time when the process appeared to have reached steady state. Therefore, the seed crystals have more driving force available to grow at the startup. After that spike, the volume mean crystal size is maintained around 82 μm. However, discontinuities are observed immediately after the cleaning events which are followed by a slight increase (1.03%) in the mean crystal size for reasons explained earlier in this section. The variation in the product crystal size is dependent on various factors. Most notably, the total number of segments. If a crystallizer is constructed of many short segments, the relative increase in the path length for the crystal flow is small. This is because a small segment is added onto the end of the reactor. If, however, the reactor is constructed of a few, large segments, then the increase in the residence time for crystals within the crystallizer may be relatively large. A five-segment crystallizer will have about a one-fifth increase in the residence time caused by a cleaning event, whereas the increase for a ten-segment reactor will be only one-tenth.

## Conclusions

In this work, a novel PFC configuration is proposed that simulates the movement of the crystallizer (hence called SM-PFC) in combination with isolation and replacement of the fouled segment during crystallization. Through simulation studies, it has been shown for a high-encrustation system that the proposed SM-PFC is able to maintain product crystal specifications (such as the mean crystal size) in the presence of heavy fouling without disrupting the process. Further, optimization of the crystallizer configuration has been carried out to find the optimum number and length of segments, temperature profile, seed rate, and volumetric flow rates which will maximize the time between cleaning events, volume mean crystal size, and productivity. A regime map has been developed for high-encrustation moderate-growth moderate-nucleation systems by collating the optimization results which can be used as a guide to design such SM-PFC systems for a given requirement such as the time between cleaning events, mean product crystal size, and productivity. For a given SM-PFC system, one can also identify the range of process outcomes which can be achieved with that crystallizer. However, in order to develop this regime map, the crystallization and encrustation kinetics need to be known. The model equations can form a stiff ODE system which would require a suitable ODE solver. Due to the nonlinearity of the optimization problem, the stochastic optimization algorithm such as the genetic algorithm has been found to provide a good balance of speed and optimization capability. Finally, the promising simulation results need to be validated experimentally. Therefore, further studies can involve building a lab scale prototype to demonstrate the effectiveness of the proposed crystallizer configuration.

## Supporting Information Available

Modeling of encrust formation, a case study discussing the performance of a PFC with a single active segment (PDF)

## Abbreviations

CSD: crystal size distribution
GA: genetic algorithm
ODE: ordinary differential equation
PBE: population balance equation
PDE: partical differential equation
PFC: plug flow crystallizer
PLC: programmable logic controller
SM-PFC: simulated moving plug flow crystallizer
TM-PFC: true moving plug flow crystallizer

## Notations

*A*_f_: flow area, m^2^
*A*_n_: annular area, m^2^
*B*_0_: nucleation rate, # m^–2^ s^–1^
*C*: solute concentration, g/g of water
*C*_b_: concentration at the bulk, g/g of water
*C*_E_: concentration at the phase boundary between encrust and viscus sublayer, g/g of water
*C*_sat_: saturation concentration, g/g of water
*c*_*p*_: heat capacity of the tube side material, J Kg^–1^ K^–1^
*c*_*p*,E_: heat capacity of the encrust, J Kg^–1^ K^–1^
*c*_*p*,W_: heat capacity of the wall, J Kg^–1^ K^–1^
*d*_p_: particle diameter, m
*D*: diffusivity, m^2^ s^–1^
*E*: activation energy, J mol^–1^
*g*: gravitational acceleration, m^2^ s^–1^
*G*: growth rate of the crystals, m s^–2^
*h*: overall heat transfer coefficient, W m^–2^ K^–1^
Δ*H*_*c*_: heat of crystallization, J mol^–1^
*k*_E_: heat conductivity of the encrust layer, W m^–1^ K^–1^
*k*_m_: mass transfer coefficient, m s^–1^
*K*_R_: surface reaction rate constant (second order), m^4^ kg s^–1^
*k*_E_: thermal conductivity of the encrust layer, W m^–1^ K^–1^
*k*_W_: thermal conductivity of the wall, W m^–1^ K^–1^
*L*: characteristic length of crystals, m
*L*_segment_: length of each segment, m
*m*: net mass of the solute deposited per unit area of encrust, kg m^–2^
*m*_d_: mass of the solute deposited per unit area of encrust, kg m^–2^
*m*_r_: mass of the solute removed per unit area of encrust, kg m^–2^
*m*_t_: mass of the solute transported to per unit area of the encrust layer, kg m^–2^
*M*_T_: magma density, kg m^–3^
*M*_W_: molecular weight of the crystal, g mol^–1^
*m*_seed_: amount of seed, wt %
*n*: crystal size distribution, # m^–2^
*N*: number of fault points within fouling layer, dimensionless
*N*_segment_: number of active segments in SM-PFC, dimensionless
feed flow rate, mL min^–1^
*r*: radial coordinate, m
*R*: gas constant, J K^–1^ mol^–1^
*R*_f_: flow radius, m
*R*_i_: inner radius, m
*R*_*o*_: outer radius, m
Re: Reynolds number, dimensionless
*t*: time, s
*t*_clean,*i*_: the time at *i*^*th*^ cleaning event, min
*T*: temperature at the tube side, °C
*T*_E_: temperature at the encrust layer, °C
*T*_W_: temperature at the wall, °C
*u*: mean fluid velocity, m s^–1^
*v*: volume of a crystal, μm^3^
*V*: volume of the crystallizer, μm^3^
*w*: local fluid velocity, m s^–1^
*z*: axial distance along the crystallizer, m

## Greek symbols

α: linear expansion coefficient, K^–1^
β: constant in growth rate expression, m
δ: encrust thickness, m
ϵ: voidage of the encrust layer, dimensionless
η: viscosity of the fluid, Pa·s
γ: constant in growth rate expression, m^–1^
λ: amplitude of oscillation, m
ϕ_*v*_: volume shape factor of the crystals, dimensionless
μ_*i*_: *i*^th^ moment of distribution, m^*i*–2^
Ω_E_: encrust domain, dimensionless
Ω_T_: tube domain, dimensionless
Ω_W_: wall domain, dimensionless
ρ_c_: density of the crystal, kg m^–3^
ρ_L_: density of the liquid, kg m^–3^
σ: relative supersaturation, dimensionless
σ_*f*_: shear strength of the encrust layer, N m^–2^
τ: residence time, s
τ_*f*_: shear stress by liquid flow on encrust layer, N m^–2^
θ: angular coordinate, dimensionless
χ: encrust thermal resistance, m^2^ K W^–1^
