# Supplementary material for: A Novel Simulated Moving Plug Flow Crystallizer (SM-PFC) for Addressing the Encrustation Problem: Simulation-Based Studies on Cooling Crystallization
Source: Ind Eng Chem Res. 2023 Mar 10;62(12):5051–64. doi: 10.1021/acs.iecr.2c02862 (PMC10064315; doi:10.1021/acs.iecr.2c02862)
Supplement: Supplementary file 1 — ie2c02862_si_001.pdf [file ie2c02862_si_001.pdf]

Supporting Information
A novel simulated moving plug flow crystallizer (SM-PFC) for addressing encrustation problem : Simulation-based studies on cooling crystallization

Aaron Bjarnason and Aniruddha Majumder*

School of Engineering, University of Aberdeen, Aberdeen AB24 3UE, UK

E-mail: a.majumder@abdn.ac.uk

Phone: +44 1224272499

Modeling encrust formation

The rate of solute transported through the boundary layer between the solution and encrust surface due to mass transfer can be modelled as

$$\frac{dm_{\mathrm{t}}}{dt} = k_{\mathrm{m}}(C_{\mathrm{b}} - C_{\mathrm{E}}); \tag{1}$$

where m_{t} is the mass of the solute transported to per unit area of the encrust layer, k_{m} is the mass transfer coefficient, C_{b} is the bulk fluid concentration and C_{E} is the concentration at the phase boundary between the encrust and viscous sublayer. Once the solute is transported, it will be integrated or deposited on the encrust layer and the rate of this disposition is given

as

$$\frac{dm_{\mathrm{d}}}{dt} = k_{\mathrm{R}}(C_{\mathrm{E}} - C_{\mathrm{sat}})^{l}; \tag{2}$$

here m_{d} is the mass of the solute deposited on the encrust layer, C_{sat} is the saturation concentration, k_{R} is the surface reaction rate constant and l is the order of surface reaction. The value of l typically lies between 1 and 2 in most of the industrial cases[1]. The reaction constant k_{R} can be found using Arrhenius approach

$$k_{\mathrm{R}} = k_{\mathrm{R0}} \exp(-\frac{E}{RT_{\mathrm{f}}}); \tag{3}$$

where k_{R0} is the reaction constant, E is the activation energy and T_{f} is the surface temperature of the encrust which can be calculated as[2]

$$T_{\mathrm{f}} = T + 0.55(T_{\mathrm{E}}\Big|_{r=R_{\mathrm{f}}(z)} - T). \tag{4}$$

In the above equation, T is the tube side temperature, T_{E} is the temperature at the encrust layer, r is the radial coordinate, R_{f} is the flow radius and z is the axial coordinate. If the reaction is controlled by mass transfer, then it can be assumed that all the solute molecules that are transported to the encrust layer will take part in reaction. This assumption is found to explain the experimental observation of encrustation reasonably well that occurs during crystallization of $CaSO_4$.[3] Therefore, we have

$$\frac{dm_{\mathrm{t}}}{dt} = \frac{dm_{\mathrm{d}}}{dt}. \tag{5}$$

The interfacial concentration C_{E} is difficult to measure and can be expressed in terms of C_b and C_{sat} with the help of eqs.(1) and (2). Following expression can be obtained for the

deposition rate

$$\frac{dm_{\rm d}}{dt} = k_{\rm m}\left[\frac{1}{2}\frac{k_{\rm m}}{k_{\rm R}} + (C_{\rm b} - C_{\rm sat}) - \left(\frac{1}{4}\frac{k_{\rm m}^2}{k_{\rm R}^2} + \frac{k_{\rm m}}{k_{\rm R}}(C_{\rm b} - C_{\rm sat})\right)^{1/2}\right]. \tag{6}$$

The order of the surface reaction is taken as $l = 2$ in the above derivation. The fraction of the deposited encrust will be subject to removal due to the interaction of the shear stress acting on the surface and the shear resistance of the encrust layer. The shear strength, σ_f, of the encrust layer is given by[1]

$$\sigma_f = K_1 \frac{P}{N\delta(1+\alpha\Delta T)d_p}, \tag{7}$$

here K_1 is a constant, P describes the intercrystalline adhesion forces, N is the number of fault points in the fouling layer, α is the linear expansion coefficient, δ is the thickness of the encrust, ΔT is the temperature difference between the wall and encrust surfaces and d_p is the particle diameter on the encrust surface. According to Bohnet[1], the following expression can be used to find the rate of removal of the solid material from the encrust layer

$$\frac{dm_{\rm r}}{dt} = K_2 \frac{\tau_f}{\sigma_f}\rho_E\left(\frac{\eta g}{\rho_L}\right)^{1/3}. \tag{8}$$

In the above equation, $m_{\rm r}$ is the mass of the solute removed per unit area of the encrust layer, K_2 is a constant, $\tau_f \propto \rho_L w^2$ is the shear stress by liquid flow on encrust layer, ρ_L is the density of the liquid phase, η is the viscosity of the liquid phase, g is the gravitational acceleration and w is the fluid velocity responsible for mixing. From eq. (8), it can be seen that the rate of removal is directly proportional to the shear stress, τ_f, and inversely proportional to the shear strength of the encrust layer. Now, substituting the expression for

σ_f from eq. (7) into eq. (8), we have

$$\frac{dm_{\rm r}}{dt} = \frac{K}{P}\rho_{\rm E}(1+\alpha\Delta T)d_p(\rho_{\rm L}^2\eta g)^{1/3}w^2\delta, \tag{9}$$

$$\frac{P}{K} = 83.2w^{0.54}, \tag{10}$$

where K is a constant. For further details of the model describing removal rate Bohnet [1] can be consulted. The net rate of addition of solute material on the encrust layer now can be found by taking the difference between the deposition and removal rates

$$\frac{dm}{dt} = \frac{dm_{\rm d}}{dt} - \frac{dm_{\rm r}}{dt}, \tag{11}$$

$$\frac{dm}{dt} = k_{\rm m}\left[\frac{1}{2}\frac{k_{\rm m}}{k_{\rm R}} + (C_{\rm b}-C_{\rm sat}) - \left(\frac{1}{4}\frac{k_{\rm m}^2}{k_{\rm R}^2} + \frac{k_{\rm m}}{k_{\rm R}}(C_{\rm b}-C_{\rm sat})\right)^{1/2}\right] - \frac{K}{P}\rho_{\rm E}(1+\alpha\Delta T)d_p(\rho_{\rm L}^2\eta g)^{1/3}w^2\delta. \tag{12}$$

Development of the encrust layer will result in increase of the thermal resistance of the PFC. The thermal resistance χ and the solid deposit per unit encrust area are related as

$$m = \rho_{\rm E}\delta = \rho_{\rm E}k_{\rm E}\chi. \tag{13}$$

where $k_{\rm E}$ is the thermal conductivity of the encrust layer. If the mass m and the encrust thickness δ are expressed as a function of thermal resistance χ, then eq. (13) can be expressed as

$$\frac{d\chi}{dt} = \frac{k_{\rm m}}{\rho_{\rm E}k_{\rm E}}\left[\frac{1}{2}\frac{k_{\rm m}}{k_{\rm R}} + (C_{\rm b}-C_{\rm sat}) - \left(\frac{1}{4}\frac{k_{\rm m}^2}{k_{\rm R}^2} + \frac{k_{\rm m}}{k_{\rm R}}(C_{\rm b}-C_{\rm sat})\right)^{1/2}\right] - \frac{K}{P}(1+\alpha\Delta T)d_p(\rho_{\rm L}^2\eta b)^{1/3}w^2\chi. \tag{14}$$

The rate of the change in thermal resistance and the rate of deposition are related as

$$\frac{d\delta}{dt} = k_{\mathrm{E}} \frac{d\chi}{dt}. \tag{15}$$

Case study: Performance of a PFC with a single active segment

A case study is presented here to investigate if the proposed SM-PFC configuration has any advantage over simpler configurations having only one active segment and one standby segment which are switched periodically at the cleaning event. The PFC is assumed to have a 10 m long active segment and a 10 m standby segment. The simulation results are then compared with the case study presented in the paper (Figure 10) where 10 active segments each having 1 m length is considered. Therefore, the total length of the active crystallizer is the same in both cases. Same operating parameters such as feed rate, temperature profile and seed mass are used. The simulation results are shown below.

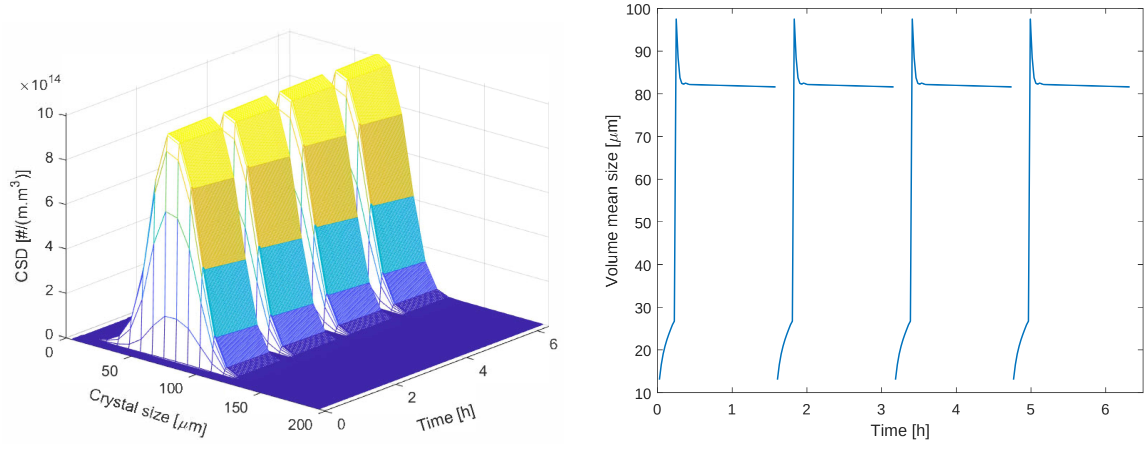

(a) (b)

Figure 1: (a) Evolution of product CSD and (b) variation in volume mean diameter for a crystallizer configuration with one active segment and one standby segment each having length of 10 m.

If we compare the simulation results for both cases presented in Figure and Figure 10

in the paper, it can be found that the volume mean size of the product crystals is similar in both cases. It is further noted that the cleaning events (denoted by the discontinuities in the CSD) are less frequent in the PFC with single active segment. However, after each cleaning event, due to the replacement of whole PFC, no product crystals are obtained for a duration of one residence time. In other words, after every cleaning event, the CSD profile is similar to starting a new operation since the whole PFC is replaced with a clean one. This has a negative impact on the productivity (reduction by 18.49%) which is down to 0.0278 kg/h for a single segment PFC while the productivity is 0.0341 kg/h for a ten segment PFC. Moreover, when there is only one long active PFC segment, the standby segment should also be of the same length (e.g., 10 m in this case) as the active segment. This is equivalent to using only half of the capacity of the crystallizer configuration at any given time. Therefore, it may not be an efficient use of resources and space. In contrast, where there are multiple active segments in the PFC, only a single fouled segment of shorter length (e.g., 1 m) is replaced by a clean one of the same length in a cleaning event. Therefore, the productivity is not much affected for the reason explained above. In summary, if a single long segment of PFC is used, similar mean crystal size can be achieved and there are less frequent cleaning events as compared to the PFC with multiple segments. However, this gain will come at a price of lower productivity and less efficient usage of equipment and space.

References

(1) Bohnet, M. Fouling of heat transfer surfaces. *Chem. Eng. Technol.* **1987**, *10*, 113–125.

(2) Coletti, F.; Macchietto, S. A Dynamic, Distributed Model of Shell-and-Tube Heat Exchangers Undergoing Crude Oil Fouling. *Ind. Eng. Chem. Res.* **2011**, *50*, 4515–4533.

(3) Brahim, F.; Augustin, W.; Bohnet, M. Numerical simulation of the fouling process. *International Journal of Thermal Sciences* **2003**, *42*, 323–334.
